# Assessing Sustainability Factors for Rural Household Sanitation Coverage in Bhutan, Kenya, Nepal, and Zambia: A Qualitative Analysis

**DOI:** 10.9745/GHSP-D-21-00724

**Published:** 2022-12-21

**Authors:** Zoe Sakas, Eberechukwu A. Uwah, Raj Kumar Bhattrai, Joshua V. Garn, Krishna Hari Gc, Anna Mutta, Kumbulani Ndlovu, Fanuel Nyaboro, Ram Prakash Singh, Ugyen Rinzin, Jedidiah S. Snyder, Kencho Wangdi, Matthew C. Freeman

**Affiliations:** a Gangarosa Department of Environmental Health, Rollins School of Public Health, Emory University, Atlanta, GA, USA.; b SNV Bhutan, Thimphu, Bhutan.; c Division of Biostatistics, Epidemiology and Environmental Health, School of Public Health, University of Nevada, Reno, Reno, NV, USA.; d SNV Nepal, Kathmandu, Nepal.; e SNV Netherlands Development Organisation, The Hague, The Netherlands.; f SNV Zambia, Lusaka, Zambia.; g SNV Kenya, Nairobi, Kenya.

## Abstract

**Background:**

Few countries are likely to achieve universal sanitation within the next decade as sustaining household sanitation coverage remains a critical challenge. This study aimed to investigate factors that may have supported or hindered sustainability of sanitation coverage 1–2 years after the completion of an integrated, area-wide sanitation program in 4 countries.

**Methods:**

We conducted qualitative analyses to identify factors related to the sustainability of sanitation coverage in Bhutan, Kenya, Nepal, and Zambia, 2 years after completion of the Sustainable Sanitation and Hygiene for All program. From November 2019 to March 2020, we conducted focus group discussions and key informant interviews with community members, project implementers, and decision makers. We triangulated the qualitative findings with data from household surveys to characterize subnational sanitation coverage throughout implementation and 1–2 years after.

**Results:**

Our data revealed behavioral, contextual, and service delivery factors that were related to the sustainability of sanitation improvements. Service delivery factors included follow-up hygiene promotion, access to construction materials, local government commitment postimplementation, functioning monitoring systems, private sector uptake of supply chain improvements, and capacity for innovation. Contextual and behavioral factors included poverty, soil type, road networks, social cohesion, desire for improved latrines, maintenance and cleaning, and knowledge of sanitation benefits.

**Conclusion:**

The presence or absence of sustainability factors identified through this research may have implications on where certain programmatic approaches will work and where adaptations may be required. By comparing sustainability factors with subnational slippage rates, we were able to illustrate how local service delivery systems may respond to barriers and enablers. Understanding the programmatic and contextual factors that either drive or hinder long-term sanitation coverage may allow for greater program impact through adapting implementation based on existing challenges in service delivery and context.

## Abstract (precis)

This study identified factors that either supported or hindered the sustainability of household sanitation coverage. The presence or absence of these factors may have implications on where certain programmatic approaches will work and where adaptations may be required.

## Teaser

**Key Findings:**

- Innovative construction approaches, local government commitment, and adequate resource allocation can help address geographic and environmental challenges, including soil type and heavy rains that cause low-quality toilets to collapse, that hinder sustainability of household sanitation coverage.
- Community health worker programs, which represent local capacity for implementing demand generation and behavior change programming, enabled the sustainability of household sanitation coverage improvements through adapting programs based on cultural norms and dynamics. Community health workers were supported by tailored trainings led by SNV and local governments.

**Key Implications:**

- Sustainable sanitation coverage requires passionate and innovative local leadership, capacity building and commitment from the local government, adaptive capacity to innovate and respond to challenges, and a strong supply chain system for sufficient resources.
- Sustainability-focused activities—such as advocating for local government involvement, guiding the local government in resource allocation, supporting reliable monitoring and evidence-based innovation, and agreeing on provision of continuous support with local partners—may extend the longevity of sanitation improvements.

## BACKGROUND

Achieving universal access to basic sanitation and hygiene remains challenging in low and middle-income countries.^1^^–^^3^ Globally, 1.7 billion people lack basic sanitation facilities and few countries are projected to accomplish universal coverage within the next decade, especially as sustainability remains a key challenge for sanitation and hygiene interventions.^4^^–^^8^ Following these concerns, understanding sustainability factors for sanitation coverage (e.g., the percentage of households with basic sanitation) has become a critical priority within the water, sanitation, and hygiene (WASH) sector.^9^^–^^10^

To address the challenge of sustaining sanitation coverage in low- and middle-income countries, interventions often aim to strengthen existing local service delivery systems through capacity building and technical assistance.^11^^–^^13^ International organizations are also starting to overtly address weaknesses within existing service delivery systems by engaging with local stakeholders including government officials, community leaders, and representatives in the private sector.^14^ Moreover, continuous political will, resource allocation, and prioritization of WASH within local governments ensure that improvements in sanitation service delivery systems are sustained postintervention.^14^^,^^15^

Understanding the programmatic and contextual factors that either drive or hinder long-term sanitation coverage may allow for greater program impact by adapting implementation based on existing challenges in service delivery and context. According to previous studies, local ownership and responsibility for sanitation services tend to support sustainable coverage, including government commitment and local capacity (e.g., resources and training) to innovate and adapt service delivery. Additionally, contextual and behavioral factors often lead to adaptations or improvements in service delivery based on the unique needs or challenges within a particular setting.^16^^–^^19^ In a recent report by the U.S. Agency for International Development, 10 contextual factors were quantitatively identified as having an impact on the sustainability of sanitation achievements, including village size, access to improved water, population density, shrubland coverage, remoteness, forest coverage, literacy, distance to water bodies, waterborne disease burden, and water scarcity.^20^ International organizations may assess existing service delivery and contextual factors through formative research to inform the design and implementation of sustainable sanitation interventions.

Understanding the programmatic, contextual, and behavioral factors that either drive or hinder sustainable sanitation coverage may allow for greater program impact by adapting implementation based on existing challenges in service delivery and context.

### Sustainable Sanitation and Hygiene for All Program Intervention

Between 2014 and 2018, the Sustainable Sanitation and Hygiene for All (SSH4A) approach was implemented by SNV, an international development a-gency based in the Netherlands, in 15 countries.^21^ The SSH4A approach focuses on 4 key components of rural sanitation programming: (1) demand creation, (2) supply chains and financing, (3) behavior change communication, and (4) WASH governance. The program was designed within the context of local government planning and budgeting to promote sustainability postintervention.^8^^,^^22^ A recent assessment of the sustainability of sanitation improvements from SSH4A programming indicated that there was sustained coverage (e.g., consistent sanitation coverage over time) in half of the program areas (Bhutan, Ghana, Kenya, Nepal, and Tanzania) and varied levels of slippage (e.g., significant decreases in sanitation coverage) in the others (Ethiopia, Indonesia, Mozambique, Uganda, and Zambia).^23^ According to this study, factors associated with sustainability through quantitative analysis of household survey data included: household socioeconomic status, baseline sanitation coverage before SNV started working in the area, and the rate of change of sanitation coverage during the program implementation period.^23^

We aimed to further explore factors that may have supported or hindered sustainability of national and subnational coverage 1–2 years after implementation. Qualitative research methods were implemented to expand on the findings from the household survey data, contextualize sustainability factors, gain a deeper understanding of how these factors impacted sanitation coverage, and elicit suggestions on how to improve sustainability. We also examined the relationship between identified sustainability factors and varying levels of slippage in SSH4A program areas using qualitative key informant interviews (KIIs) and focus group discussions (FGDs) with in-country project stakeholders, implementers, and community members.

## METHODS

We conducted qualitative analyses to identify factors related to the sustainability of sanitation coverage in 4 countries (Bhutan, Kenya, Nepal, and Zambia) 2 years after completion of SSH4A activities in select program areas.

### Country and District Selection for Case Studies

This study was nested within an evaluation of a 5-year multicountry sanitation program. As part of this evaluation, repeated cross-sectional household surveys were administered throughout implementation and 1–2 years after the intervention was completed to assess the sustainability of sanitation coverage gains. Details of the purpose and methods for this evaluation and the quantitative analyses are published elsewhere.^23^ Briefly, a multistage cluster sampling scheme was used to select a random and representative sample of 12 program areas in 10 countries. Data were collected on household WASH access and use, including direct observations of sanitation facilities. For this study, we aggregated the household data at the district level to show district-level sanitation trends over time. Our study team performed analyses to assess the impact and sustainability of key household WASH variables. These quantitative findings were used both to select study sites and to inform our qualitative findings.^8^^,^^23^

We assessed impact and sustainability of key household WASH variables and used these findings to select study sites and inform our qualitative findings.

We selected program areas from 4 of the 10 countries for further qualitative research. Research was conducted in Sakteng and Udzorong regions in Bhutan; Homa Bay and Kericho regions in Kenya; Siraha region in Nepal; and Kasama and Mporokoso regions in Zambia. We chose countries and regions based on diversity of sanitation facility coverage improvements; postintervention slippage; and qualitative factors including cultural diversity, implementation variety, toilet quality, national policies, and geography.^8^^,^^23^ Selection of regions and targeted locations for qualitative data collection was also influenced by availability of local research assistants from SNV and practical considerations such as transportation. Table 1 outlines geographic factors, population density, and livelihood in each of the included areas.

**TABLE 1.** Observed Contextual Factors From 4 Rural Sanitation Sustainability Case Studies in Bhutan, Kenya, Nepal, and Zambia

	**Bhutan**		**Kenya**		**Nepal**	**Zambia**	
**Geographic Area**	**Sakteng** ^a^	**Udzorong** ^a^	**Homa Bay** ^b^	**Kericho** ^b^	**Siraha**	**Kasama**	**Mporokoso**
Geographical features	High altitude; clustered houses; cold climate	Steep hills; moderate weather	Rocky as well as collapsible soils; high water table Suba South: near lake	Rocky and collapsible soil; accessible roads	Fertile; moderate weather; occasional flooding	Clay and sandy soil; frequent rain; limited space (clustered houses)	Heavy clay soil; fertile; heavy rains
Population density (remote, rural, or periurban)	Rural; accessible roads to town centers	Rural; villages connected through farm roads	Rural or periurban (depending on the village)	Rural or periurban (depending on the village); small hills	Periurban; rural	Periurban; near a large town	Lunte: remote, very rural with poor road network
Livelihood	Animal husbandry; migratory populations	Subsistence farming; cattle	Farming; cattle; fishing	Coffee; tea; farming on plantations	Farming; animal husbandry	Small businesses; selling in markets	Chisha-mwamba: farming compounds

a Gewogs within the Trashigang district of Bhutan.

b In Kenya, data were collected in Rangwe and Suba South subcounties within Homa Bay county; and in Belgut and Kipkelion subcounties within Kericho county.

### Data Collection

We collected qualitative data, including KIIs and FGDs, from November 2019 to March 2020 in Kenya, Nepal, and Zambia at the national, subnational, and community levels. Data collection in Bhutan was delayed due to the coronavirus disease (COVID-19) pandemic. When the travel and research restrictions were lifted in December 2020, we held KIIs and FGDs with additional precautions, including social distancing, prioritizing outdoor locations, and offering masks and sanitizer. We summarize data collection activities in Table 2.

**TABLE 2.** Summary of Qualitative Data Collection Activities and Participant Characteristics From 4 Rural Sanitation Sustainability Case Studies in Bhutan, Kenya, Nepal, and Zambia

	Bhutan, No.	Kenya, No.	Nepal, No.	Zambia, No.	Total, No.
Interviews conducted	13	18	23	18	61
SNV staff	2	4	2	2	10
National government	2	2	2	1	7
District government	0	3	3	5	11
Subdistrict government	4	9	4	3	20
Village	2	1	7	6	16
Local nongovernmental organization	0	0	4	0	4
Private sector	3	3	2	5	13
Total interview participants	13	22	23	22	80
Focus groups	8	12	10	10	40
With women	4	6	5	5	20
With men	4	6	5	5	20
Focus group participants	79	89	82	98	348
Aged 55 years and older	19	25	15	29	88
Persons with disability	2	9	1	6	18

Interview guides were adjusted to local contexts with input from research assistants working for each SNV in-country team.^24^ Guides were translated into local languages when necessary. When appropriate, interviews were conducted by the lead researcher in English. We conducted a total of 61 interviews with 80 key informants. Interviews included 1 or 2 key informants and lasted 45–90 minutes. National-level interviews were conducted with government officials and representatives from international organizations and development agencies. Subnational-level interviews were conducted with public health officers, community health workers, masons, and community leaders.

Program staff conducted FGDs (N=40) with community members at the village level. All community FGDs were held in local languages identified by local research assistants. Each FGD included 6–12 participants and lasted approximately 2 hours. Demographic information was collected after each FGD. To protect participant confidentiality, we did not attribute specific demographics to each participant, and therefore did not organize quotes and feedback by these groups. Collecting the demographic information allowed us to ensure an inclusive sample.

With permission from participants, we audio-recorded KIIs and FGDs, except for some national-level interviews where we relied on notes for analysis. Recordings were transcribed and translated into English. All recordings and transcriptions were password-protected and uploaded to a secure folder. We conducted daily debriefs with the lead researcher and all research assistants to discuss emerging themes, follow-up questions, and potential iterations of the topic guides.

### Tool Development

Data collection tools, including topic guides and demographic surveys, were developed primarily by our research team. We collaborated with SNV in-country staff to ensure that topic guides sufficiently probed for previously identified barriers and enablers and to confirm that all data collection activities were culturally appropriate and acceptable.

### Desk Review

To inform tool development and conceptual framing, we conducted a thorough desk review of SNV program documents to further understand the SSH4A approach and completed a comprehensive literature review of rural sanitation methodologies. We also evaluated key findings from SNV’s performance monitoring data which measured improvements in sustainable programming.^21^^,^^24^ Table 3 outlines the 4 components of the SSH4A approach, expected outcomes, and indicators to measure success and sustainability throughout implementation.

**TABLE 3.** Sustainable Sanitation and Hygiene for All Approach Components and Outcome Indicators

**Program Components**	**Expected Outcome**	**Outcome Indicators**
Demand creation	Local organizations are capable to implement and steer sanitation demand creation at scale with quality.	Progress in the capacity of local government or line agencies to steer sanitation demand creation processes with quality in their area.
		Progress in capacity in the area to implement sanitation demand creation with quality.
Sanitation supply chains and finance	Appropriate market-based solutions for a variety of sanitation consumer needs are implemented at scale.	Progress in private sector engagement in sanitation hardware and services.
		Progress in availability of affordable sanitation options for the poorest wealth qualities.
Behavior change communication	Progress in the commitment and capacity of local organizations to implement behavior change communication with quality.	Progress of responsible line agencies to institutionalize BCC for sanitation and hygiene.
		Progress in the capacity of staff to implement improved practice in BCC for sanitation and hygiene.
		Progress in local multistakeholder rural sanitation and hygiene sector alignment.
		Progress in the capacity of agencies to proactively mainstream gender and social inclusion in rural sanitation and hygiene services.
WASH governance	Improvements in local WASH governance in terms of alignment of stakeholders, sector planning and monitoring, transparency, and social inclusion.	Progress in the capacity of local government to provide sustainable social support mechanisms for rural sanitation and hygiene.
		Progress on the influence of women in rural sanitation and hygiene programs.
		Progress on the influence of poor households in rural sanitation and hygiene programs.
		Progress on the influence of people with disabilities in rural sanitation and hygiene programs.

Abbreviations: BCC, behavior change communication; WASH, water, sanitation, and hygiene.

### Multicountry Workshop

**FIGURE 1.**
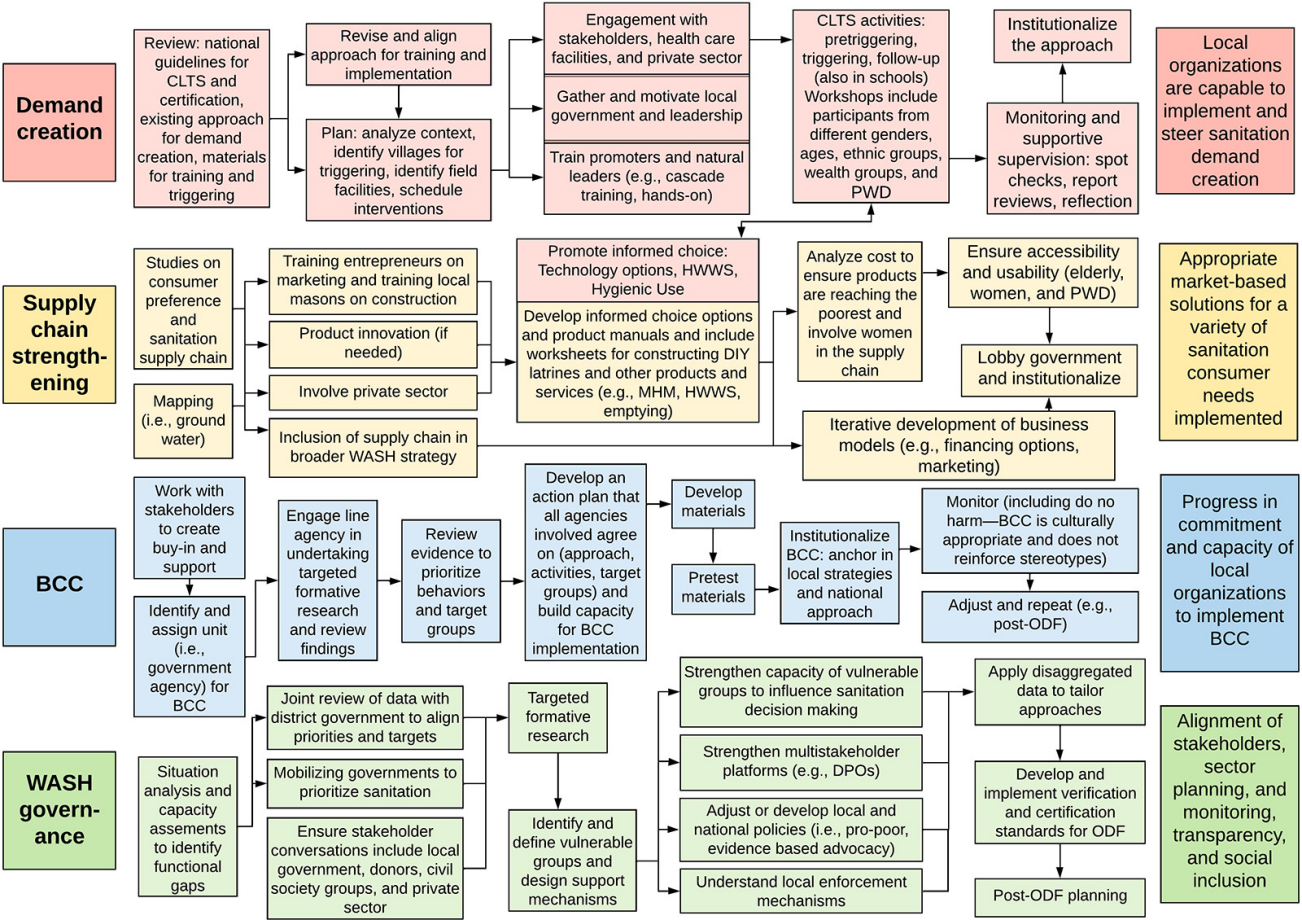
SSH4A Programmatic Implementation Process Flow Chart Reconstructed by SNV Program Staff From 11 Countries in Africa and Asia Abbreviations: BCC, behavior change communication; DIY, do-it-yourself; DPO, Organisations of Persons with Disabilities; CLTS, community-led total sanitation; HWWS, handwashing with soap; MHM, menstrual hygiene management; ODF, open defecation free; PWD, persons with disabilities; SS4HA, Sustainable Sanitation and Hygiene for All.

Before formal data collection began, in August 2019, we held a workshop with program managers, implementers, and stakeholders at the SNV learning event in Accra, Ghana, to understand the SSH4A approach from the implementers’ perspectives. Participants included program staff from 11 countries (Bhutan, Burkina Faso, Ethiopia, Ghana, Kenya, Laos, Nepal, Rwanda, Tanzania, Uganda, and Zambia). Together, we reconstructed the SSH4A implementation process to highlight program components, outputs, and outcomes across a variety of contexts. Because KIIs and FGDs were held 1–2 years postintervention, it was important for our research team to collaborate with SNV staff to better understand activities implemented through the SSH4A approach. The implementation process flow chart provides details about the activities conducted or supported by SNV (Figure 1). Data collection and analysis tools, including topic guides and codebooks, were informed by the information gained from this workshop as we asked participants to discuss how capacity-building activities implemented by SNV strengthened service delivery and to identify gaps postintervention. We introduced key themes, activities, and potential sustainability factors that emerged from this workshop through probes in topic guides and as codes in analysis.

### Qualitative Data Analysis

**FIGURE 2.**
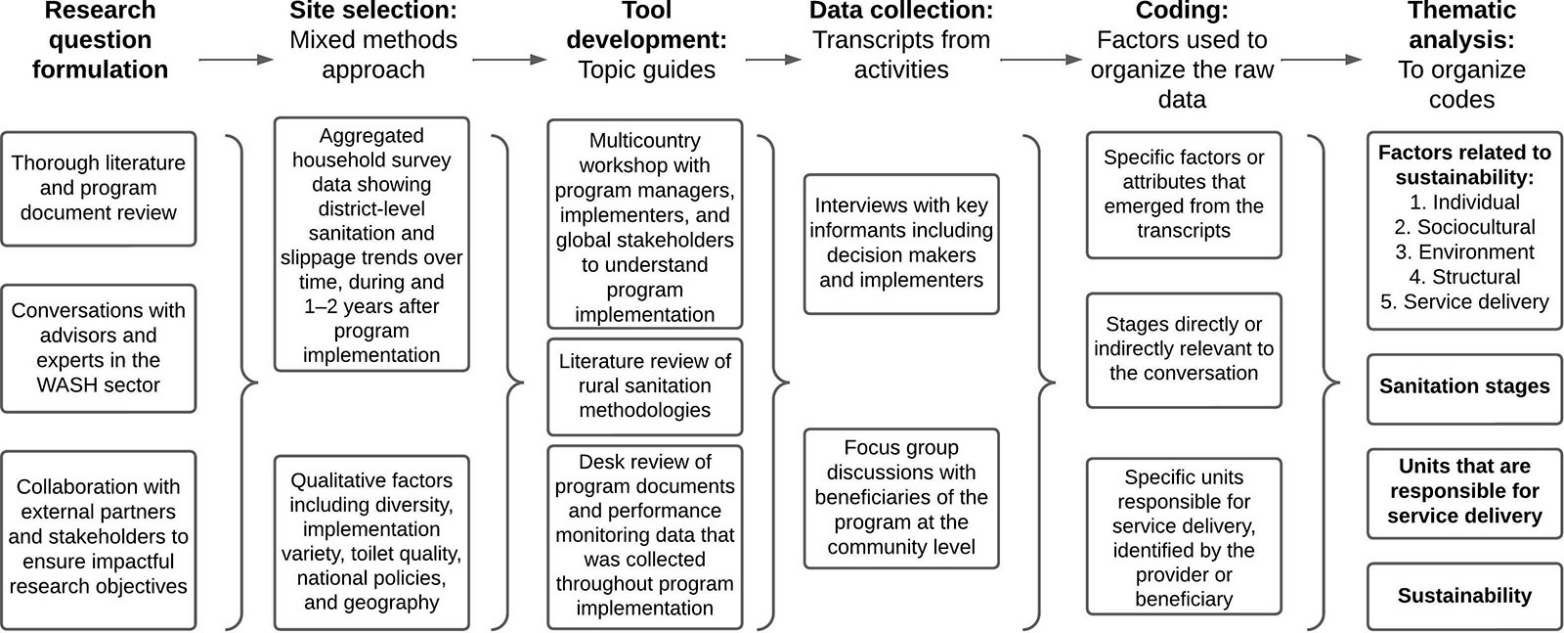
Data Analysis Process and Definitions of Key Terms for Qualitative Research on Rural Sanitation Coverage Abbreviation: WASH, water, sanitation, and hygiene.

We conducted inductive and deductive thematic analysis to identify factors related to the sustainability of sanitation improvements. The qualitative codebook was developed using a deductive approach, applying ideas from existing rural sanitation frameworks that focused on institutionalization of services, policies, governance, capacity building, financing, infrastructure, and monitoring. The Ottawa Charter for Sanitation Stages framework combines service delivery factors (e.g., affordability of latrines and acceptability of messaging) with behavioral, environmental, cultural, and structural factors related to sustainable sanitation.^25^ We primarily applied factors from this framework, with additions from other frameworks as needed to appropriately categorize the data. Inductive codes were added to account for emerging themes. All analyses were conducted using MAXQDA software. The data analysis process and codebook are outlined in Figure 2 and Table 4 (the fifth column in Figure 2, titled “coding,” corresponds with the codebook described in Table 4). The comprehensive codebook and topic guides for KIIs and FGDs are available on our Open Sciences Framework webpage.^24^

**TABLE 4.** Summary of the Codebook Used for Analysis^a^

**Category**	**Definition**	**Examples of Codes**
Individual	Factors related to individual processes (i.e., psychological or behavioral) and intrahousehold dynamics	Perceived cost, decision making, ownership/pride, education, privacy
Social and cultural	Factors related to social and cultural perspectives, including values, beliefs, and interactions between community members	Cultural norms, traditional leadership, social aspect of disease, enforcement of bylaws
Environmental	Factors related to the physical environment	Geography (soil structure), climate, quality and design of toilets, pit design, space availability
Structural	Factors related to the economic, political, and institutional aspects of sanitation	Institutionalization of services, poverty and social class, government financing, equity, tenancy
Service delivery	Factors related to sanitation service delivery from development partners, local government, private sector, and community stakeholders	Demand creation and behavior change, follow-up hygiene promotion, supply chain, stakeholder engagement, training, monitoring
Responsible units	Responsible units for each particular service delivery factor; associated with service delivery codes	Local government, development partners (iNGOs), local NGOs, private sector, traditional leaders, CHWs
Sanitation stages	Five stages of sanitation coverage and usage	Acceptance, construction, utilization, maintenance (including reconstruction), safely managed sanitation
Sustainability	Sustainability of access and use of toilets; used whenever the participant discusses sustainability, long-term access or use, continuity, or longevity of services	N/A

Abbreviations: CHW, community health worker; iNGO, international nongovernmental organization; N/A, not applicable; NGO, nongovernmental organization.

a Adapted from the Ottawa Charter for Sanitation Stages framework.^25^

### Ethical Approval

The Institutional Review Board of Emory University deemed the study exempt from review. All respondents gave their written or oral consent to participate in the study. The research project and data collection activities were also approved by local government entities in all 4 countries where data were collected.

## RESULTS

### Sanitation Coverage and Sustainability Across 4 Countries

Rural sanitation improvements were generally sustained in Bhutan, Kenya, and Nepal, but there were higher levels of slippage in Zambia.^23^ Table 5 illustrates historical and current rural sanitation coverage and slippage levels in the 26 selected districts for this study. In Zambia, because of the large population size and significant differences in sanitation coverage between subdistrict areas (e.g., wards), we conducted an additional ward-level analysis to ensure qualitative data were collected in diverse locations (data not shown). We found that there was no recorded slippage in Chishamwamba (+4%) and increased slippage in Lunte (−17%), 2 wards in Mporokoso, and high levels of slippage in Chiba (−61%) and Lua-Luo (−52%), 2 wards in Kasama. These localities are referred to in the qualitative results.

**TABLE 5.** Sanitation Coverage and Slippage Rates^a^ for SSH4A Program Areas^23^

Country	Geographic Area	Population at Endline	Baseline, %	Endline, %	Postintervention, %	Percentage Point Change, %
Bhutan	Trashigang^b^	35,410	57	95	97	+2
	Samtse^c^	59,701	64	90	--	--
Kenya	Belgut^d^^,^^e^	77,813	16	85	88	+3
	Kipkelion^b^^,^^e^	26,967	8	94	81	−13
	Rangwe^b^	129,032	34	67	66	−1
	Suba South^b^^,^^e^	127,901	25	66	58	−8
	Kaloleni	100,205	15	53	55	+2
	Keiyo	87,101	11	80	77	−3
	Magarini	102,706	16	32	44	+12
	Malindi^e^	27,891	16	63	49	−14
	Marakwet	51,040	22	88	86	−2
	Sigowet Soin	86,097	14	70	74	+4
Nepal	Siraha^b^^,^^f^	64,423	7	94	94	0
	Banke^f^	104,540	6	87	92	+5
	Dailekh^f^	131,018	56	94	89	−5
	Humla^f^	24,080	23	93	100	+7
	Jumla^g^	38,383	56	94	95	+1
	Mugu^e^^,^^f^	31,799	46	100	89	−11
	Saylan^g^	81,869	64	100	98	−2
	Saptari^f^	120,828	1	95	98	+3
	Sarlahi^g^	95,745	2	100	98	−2
	Surkhet^f^	71,735	62	100	100	0
Zambia	Kasama^b^^,^^e^	200,588	8	91	74	−16
	Mporokoso^b^^,^^e^	123,412	18	95	88	−6.5
	Mungwi^e^	174,412	5	83	50	−33
	Luwingu^e^	175,116	15	96	82	−15

Abbreviations: COVID-19, coronavirus disease; SS4HA, Sustainable Sanitation and Hygiene for All.

a Sanitation coverage refers to the percentage of households with latrines. Slippage refers to the decrease in sanitation coverage overtime. More information available in Apanga et al.^23^

b Selected for qualitative research.

c Data from Samtse are not available due to delays in data collection from COVID-19.

d District boundaries changed during our study period, which may impact the accuracy of data.

e District with elevated slippage.

f Nepal 2 SSH4A program area.

g Nepal 1 SSH4A program area.

Rural sanitation improvements were generally sustained in Bhutan, Kenya, and Nepal, while there were higher levels of slippage in Zambia.

**FIGURE 3:**
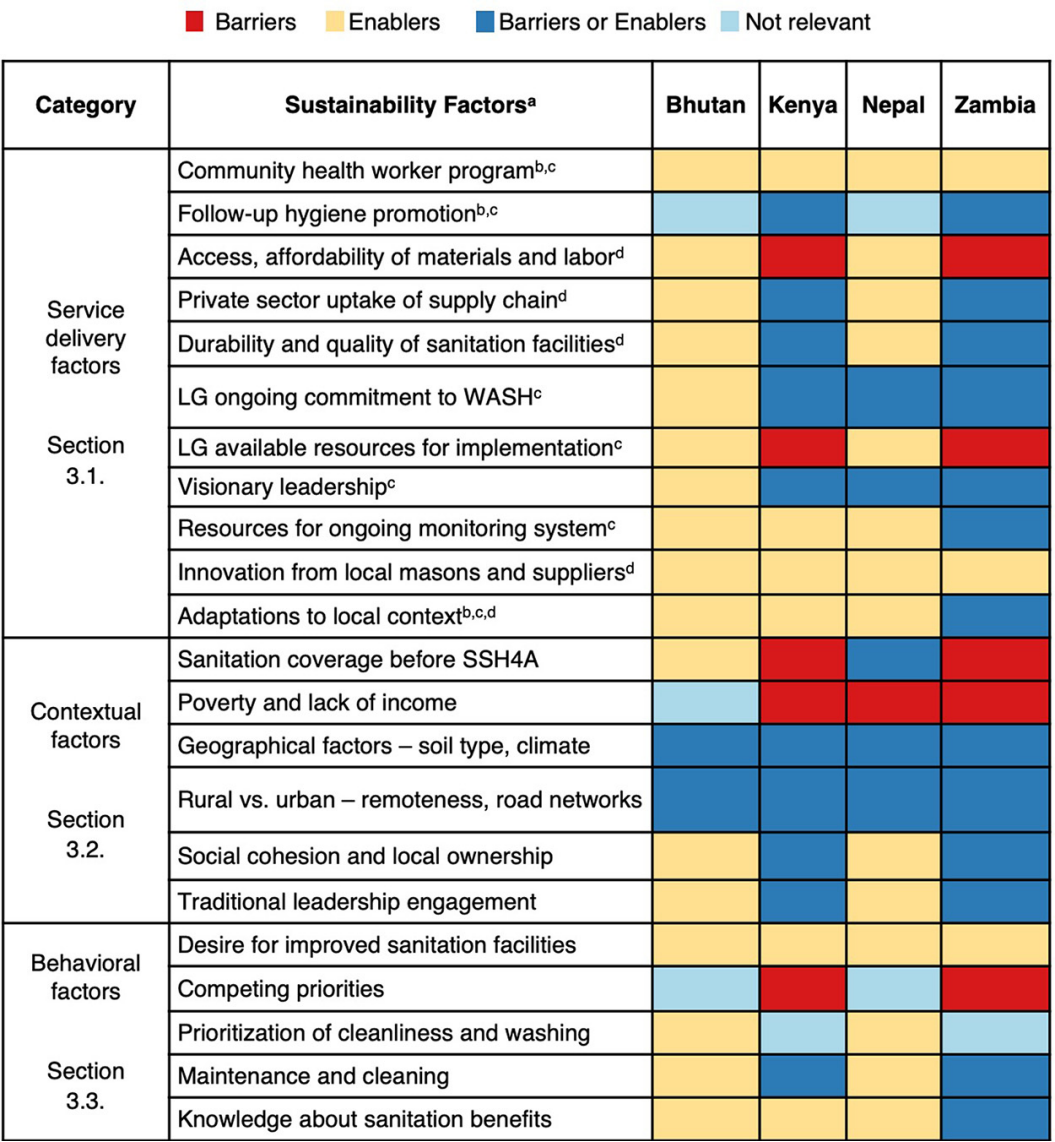
Heat Map Illustrating Sustainability Factors for Rural Sanitation Coverage LG, local government; SS4HA, Sustainable Sanitation and Hygiene for All; WASH, water, sanitation, and hygiene. ^a^ Superscript notes indicate related components of the SSH4A approach. ^b^ Demand creation and behavior change. ^c^ Governance. ^d^ Supply chain strengthening and finance.

We derived the findings on sustainability factors from analysis of data from KIIs and FGDs. The heat map compares sustainability factors across countries assessed in this study (Figure 3). The sustainability factors are separated into 3 categories: (1) service delivery, which refers to components of the local service delivery system; (2) contextual, which refers to environmental and social factors; and (3) behavioral, which refers to individual factors related to decision making. Sustainability factors can be described as barriers (hindering sustainability) or enablers (driving sustainability). For example, in Kenya and Zambia, lack of access to materials was typically discussed as a barrier to sustainability, while in Nepal and Bhutan easy access to materials was discussed as an enabler. In all 4 countries, geographical factors were discussed as either barriers or enablers, depending on the location within the country. For example, areas with collapsible soil or frequent flooding discussed barriers, and areas with flat land and clay-like soil for brick construction discussed enablers.

### Service Delivery Sustainability Factors

**FIGURE 4.**
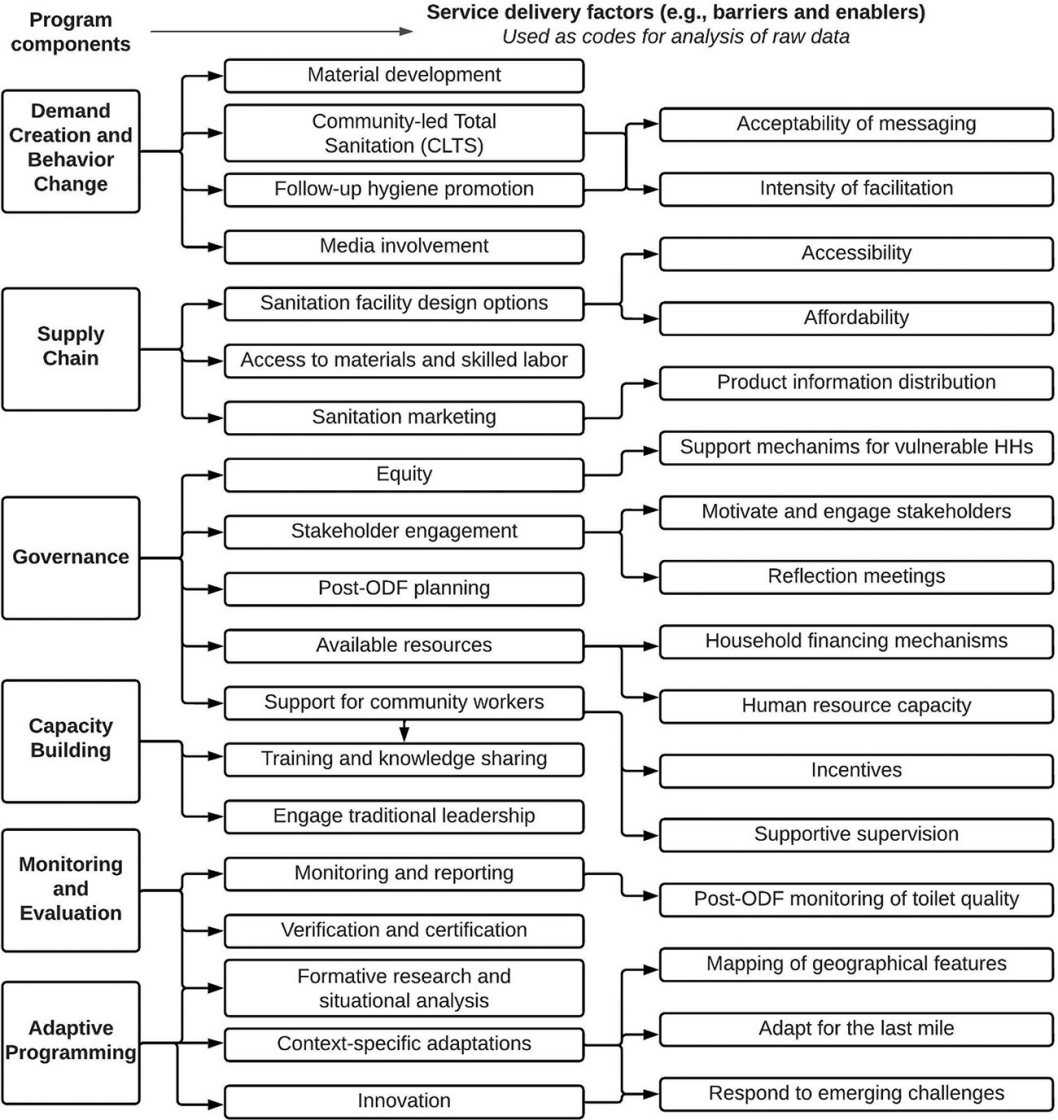
Service Delivery Factors: Coding Tree From Analysis of Raw Data for Qualitative Research on Rural Sanitation Coverage in Bhutan, Kenya, Nepal, and Zambia Abbreviations: HH, household; ODF, open defecation free.

We grouped service delivery sustainability factors into categories that emerged from the data: (1) demand creation and behavior change; (2) supply chain and finance; (3) governance; (4) capacity building; (5) monitoring and evaluation; (6) adaptive programming (Figure 4).

#### Demand Creation and Behavior Change

Community members and government officials primarily described community health worker programs as enablers for sustainability because they represent local capacity for implementing demand generation and behavior change activities. Community health workers’ ability to adapt to social and cultural norms relevant to the areas where they work and live may also support sustainable programming. SNV, with support from local governments, typically led trainings for community health workers, volunteers, and traditional leaders.

*The most important thing is door-to-door outreach, where you talk to people at individual level to understand why they don’t have a latrine, or why they have a latrine but they are not using them.* —SNV staff, international NGO, Kenya

Continuous follow-up for health promotion was also discussed as an essential aspect of sustainable service delivery. Consistently reminding community members about the importance of latrine construction, use, and maintenance may be required months, even years, after a community is considered open defecation free (ODF). Additionally, tailored messaging for households who need to reconstruct latrines after they have collapsed likely helped to maintain coverage in areas where lower latrine quality was more common.

*People want to see us many times. That is when they realize it is serious. If you sensitize them and then you forget about them, they also forget about the whole thing. When they see us often, it sticks more. —*Community-led total sanitation coach, government employee, Zambia

#### Supply Chain and Finance

Access to construction materials (e.g., plastic, cement, culverts, and bricks) and skilled labor varied between and within countries and was identified as a sustainability factor by all key informants (N=80). Lack of access to materials was most frequently reported by community members in rural areas. In Kenya and Zambia, key informants commonly mentioned that lack of access to materials was a challenge for constructing durable latrines. This lack of access may be due to affordability of materials, poverty and lack of income, or poor road networks to central urban locations.

*You will find that lack of finances to buy [cement] leads people to build the other pit latrines that we have been talking about that collapse in [less than] 2 years. We don’t have enough resources to build permanent latrines that won’t collapse.* —Woman, community member, Lua-Luo, Kasama, Zambia

However, most FGD participants also discussed the use of locally available materials, including logs and bricks made from clay-like soil, which were more accessible.

*Most of the toilets here, when we build them they last many years and the reason why they last many years is because the soil here is like clay. Therefore, the toilets do not collapse inside so the latrines last very long because the soil is strong.* —Community leader, Chishamwamba, Mporokoso, Zambia

In Nepal and Bhutan, these issues were not as prevalent, and access to affordable materials for durable latrine construction was considered an enabler in some areas. For example, in Bhutan, the Forest Park Office (a government entity) provided free timber for sanitation facility construction.

*When people raised the concern of not having access to timber to build toilets, we also had a strategy for close collaboration with the Forest Park Office to allocate free timber to every household, which facilitated the construction of toilets. I think the results we achieved have been mainly due to close collaboration and teamwork between [local] leaders, the Ministry of Health, and the Forest Park Office. —*Community leader, Trashigang district, Bhutan

Key informants, especially district-level government staff and active masons, also discussed uptake of supply chain improvements by the private sector. In all program areas, key informants reported that SNV trained masons to construct toilets, adapt construction and design to account for geographical factors and individual preferences, and market their products to consumers.

*SNV once brought a project to build toilets with masons, so they discussed with us how these toilets will be built and we had different prices for these toilets based on how much one could manage to pay… I personally joined this group. —*Woman, community member, Lua-Luo, Kasama, Zambia

SNV trained masons to construct toilets and adapt construction and design to account for geographical factors and individual preferences.

During training, SNV also prioritized accessibility of toilet design for the elderly and persons with disabilities. This included handrails and raised toilet seats.

*SNV has also made a manual about how to make disabled friendly toilets with options. We talked about it during mason training.* —Local NGO staff, Nepal

In Kenya and Zambia, masons reported that they were working at the district level (often headquartered in the main town) and traveled to many communities for work, making very remote areas unappealing for business. One mason from a remote community, who was trained by SNV, claimed he was unable to work as a mason because he could not access the required materials. The village he lived in was on a dirt road that often flooded and was more than a 3-hour drive from the closest mid-sized town. In Bhutan, where collaboration between SNV and the national government was particularly seamless, these trainings were led by the local health sector with support from SNV staff. Although the success of implementation varied between countries, all of the masons that were interviewed (n=13), in all 4 countries, discussed the knowledge and skills gained from training led by SNV.

*The government health sector invited me to a meeting and that gave me the opportunity to be involved in sanitation business. In town, there was already an entrepreneur who was doing sanitation business. And there was no one running a sanitation business in my [rural] area, despite having a bigger population. The officials asked me if I could be a supplier… I agreed to supply all kinds of materials like toilet pots, pans, pipes, tin sheets, and even the plumbing equipment. This initiative from the government helped me a lot in my business and even benefitted the households as people do not have to travel to far places to buy sanitation materials.* —Small-medium enterprise, private sector, Trashigang district, Bhutan

Key informants also mentioned additional sanitation marketing activities, including distribution of product manuals to promote informed choice of facilities, advertisement of improved toilets in schools and public spaces, and consumer research to identify preferences. However, sanitation marketing initiatives diminished in some areas, according to informants, without continuous support from SNV.

*If somebody wants to have their latrine improved, [SNV] had formed a [sanitation marketing] committee [which would assist with in-kind payment options]. But when they went to the local authority to get the [materials] they use because of the transfers, it couldn’t work out because today they talk to 1 person, and then they were transferred, and so it became so difficult.* —Woman, community member, Kasama, Zambia

Strength of the existing supply chain, uptake of supply chain improvements by the private sector, affordability of materials and labor, and socioeconomic factors all had an impact on the durability and quality of sanitation facilities. In Kenya and Zambia, some areas had higher-quality latrines (Belgut, Rangwe, and Chishamwamba), while others had very poor-quality latrines, commonly referred to as “temporary” latrines (Suba South, Kipkelion, and Kasama). Participants from communities with lower-quality latrines discussed the difficulties associated with frequent reconstruction, including finances, time, and lack of motivation to reconstruct.

*If you visit a household and they don’t have a latrine, you will ask them to build it using locally available materials, but as time goes maybe the locally available material actually fades, and they have not changed them into a better latrine, so they will stop using it especially the children because they will fear they could fall inside.* —Local government staff, Belgut, Kericho, Kenya

Participants from communities with lower-quality latrines discussed the difficulties associated with frequent reconstruction, including finances, time, and lack of motivation to reconstruct.

In Nepal and Bhutan, most latrines were durable, regardless of climate, as they were made from strong materials. In Nepal, participants in all FGDs (n=10) reported that no one in their community needed to reconstruct and described that all toilets in their area were built with either cement rings (e.g., culverts) or septic tanks.

*There are 4 or 5 types of toilets. Pit latrines are 1 option, but they are not safe. Mainly, we can build 2 kinds of toilets: toilets with septic tanks and toilets with cement rings are found in our community.* —Woman, community member, Mahadeva, Siraha, Nepal

#### Governance, Capacity Building, and Monitoring and Evaluation

According to government officials in all 4 countries, the SSH4A approach invested considerable time and resources in strengthening sustainable governance structures through capacity building and technical support. However, the extent to which local governments maintained sufficient financial and human resources for sanitation activities varied between districts and countries. Ongoing commitment from local governments after SSH4A implementation was commonly cited as an important sustainability factor, and areas that lacked local government commitment and resources might have experienced more slippage. However, there was a clear distinction between commitment from the implementation level (e.g., district, ward, or municipality offices) and commitment from the national level. Especially in Kenya and Zambia, there were health officers at the district or ward level that were highly committed to improving sanitation in their area, but the lack of national-level government commitment resulted in a lack of financial and human resource allocation to the district officers for sanitation activities.

*I think there was a bit of relaxation during SNV’s handover, such that things couldn’t continue like the way they were done previously, at the pace it started with. What is sad is that, earlier on, even up till now, when the program was being conducted, not all clinic facilities had environmental health technologists. The Environmental Health Technicians [EHTs] are responsible for the continuation of sanitation activities in catchment areas…For it to continue, there is a need for capacity building of the EHTs who are in those health facilities so that they continue with the behavior change activities.* —Environmental health officer, government staff, Zambia

Local governance systems were also augmented by post-ODF planning (e.g., sustainable behavior change, maintenance, and fecal sludge management), continuous training and engagement with community workers, and attention to equity. Data also suggested that community members who were aware of their local government’s commitment to sanitation were more inclined to prioritize sanitation themselves. Related is the local governments’ ability to support continuous training and engagement for community workers, traditional leaders, and new staff members and to support monitoring and evaluation activities once external funding and support conclude.

*There was a royal visit to Sakteng, and His Majesty the King said that he will not accept anything but good sanitation, hygiene, and health from all of us, the people of Sakteng… this has inspired and motivated us to improve our sanitation and hygiene. Since then, every month, we have met and started improving. And the local health assistants, park officials, and even police officers continue to follow up and support identifying locations for toilets and provide other technical advice.* —Man, community member, Sakteng, Trashigang, Bhutan

Data suggested that community members who were aware of their local government’s commitment to sanitation were more inclined to prioritize sanitation themselves.

#### Adaptive Programming

The capacity of local governments, private sectors, and local nonprofit organizations to adapt or alter programmatic activities and sanitation service delivery after SSH4A implementation varied between program areas. Adaptive capacity may be affected by training facilitated by the development partner, resources allocated for reflection and evaluation, the presence or availability of people who have the skills needed to innovate and respond to challenges, and employee turnover in local health offices. Two components of adaptability that were most frequently mentioned by key informants were local masons’ ability to innovate on toilet designs and local decision makers’ ability to adapt to contextual changes or specific needs. For example, in areas with masons who were innovative and could adapt toilet design to withstand geographical challenges, sustainability of individual latrines was more common.

### Contextual Sustainability Factors

Contextual sustainability factors are not direct objectives of sanitation interventions or activities and may not be able to be altered or improved through WASH interventions but are still identified by key informants as being related to the sustainability of sanitation improvements. These factors are usually considered when designing sanitation programs because adaptations or innovations may be required to address contextual barriers or enablers. For example, in areas with thick, clay-like soil, community health workers may teach individuals how to build their own superstructures out of locally made bricks. However, in areas with collapsible soil, supply chain strengthening to support the development of sanitation technologies that are both durable and affordable may be more appropriate.

Table 6 summarizes the influence of contextual sustainability factors on sanitation service delivery and how these contextual factors may have a positive, negative, or neutral impact on service delivery factors identified by key informants.

**TABLE 6.** Impact of Contextual Factors on Sanitation Service Delivery

**Contextual and Behavioral Factors**	**Impact on Service Delivery Factors**
Poverty and income availability	Latrine options, household financing, subsidies
Gender roles	Maintenance responsibility, target for demand creation
Marginalized groups and defiant groups	Tailored messaging, enforcement from traditional leaders or local government
Soil type and climate	Supply chain, toilet design, sanitation marketing, messaging for reconstruction, monitoring consistency
Rural vs. urban, remoteness	Tailored messaging to adapt to cultural differences, accessibility of materials, toilet design, tenancy considerations, how fecal sludge is safely managed
Space availability	Toilet location in relation to the house, quality and durability, how fecal sludge is safely managed
Social cohesion and values	Demand creation activities, tailored messaging

#### Poverty and Lack of Income

Poverty was identified as 1 of the most pressing concerns related to the sustainability of sanitation coverage from the perspectives of our key informants, especially in Kenya and Zambia. Lack of income (in some areas, lack of any available cash) prevented community members from investing in durable latrine options because they could not afford high-quality materials (e.g., cement, culverts, and bricks) or the labor required to dig strong, deep pits that would not collapse or fill up quickly.

*People love durable toilets but fail to raise the money to pay [for them]. People appreciate the improved kinds of toilets that we build, it’s just that they don’t have money to afford.* —Mason, private sector, Mporokoso, Zambia

Key informants identified poverty as a most pressing concern related to sustainability of sanitation coverage, especially in Kenya and Zambia.

Consequently, lack of affordable high-quality materials may also be due to underdeveloped markets, especially in African countries, which further exasperates this inequity. The cost of cement, slabs, and culverts in Zambia and Kenya was considered expensive by most participants.

*Financial problems can be a challenge, I want to build [my latrine] with cement, I want to buy a slab for it, but these other temporary latrines, we just build them because we don't have income [to pay for durable materials] … if income was here, we could construct the long-lasting ones, me that's my opinion.* —Woman, Community member, Rangwe, Homa Bay, Kenya

In most areas, formal household financing mechanisms were scarce or unavailable. However, community savings groups and in-kind payments to masons offered an opportunity for households in the lowest wealth quintiles to invest in sanitation facilities.

*You can get some money from a savings group, then it becomes a loan because you have to pay back with interest. So as a community, we do not get loans from the bank, but from savings groups.* —Man, community member, Chishamwamba, Mporokoso, Zambia

In Nepal, communities who self-identified as poor still claimed they would construct “good toilets” through loans or support from the community and that they would construct high-quality toilets even though they were poor. Overall, community members in Nepal reported that open defecation was “not an option” regardless of socioeconomic challenges.

*Poor people who have no money and who have no one in their household to make money, they have taken loans out to build toilets, because there is no option of building a toilet. One must make toilet in our community.* —Man, community member, Siraha, Nepal

#### Geographical and Environmental Challenges

Geographical barriers, including sandy or rocky soil, high water table, heavy rain, and flooding were mentioned in all countries but not in all program areas. Typically, these barriers seemed more intrusive to participants who used lower-quality latrines rather than durable toilets. Community members and masons working in the private sector were most concerned about environmental challenges associated with latrine construction.

*Slippage happens during rainy season when the floods happen, most of the toilets collapse and coverage goes down and people need to start [building a latrine] again.* —National-level government staff, Homa Bay, Kenya

Geographical barriers were more intrusive to participants who used lower-quality latrines rather than durable toilets.

Innovations were applied to adjust to these geographical barriers, including raised toilet platforms to avoid flooding pits and building toilets inside to prevent destruction from winds. However, innovations were more common in areas with durable latrines because use of locally available materials does not provide as much flexibility in terms of facility design.

*There are toilets which are built outside of houses. Weather could affect and become a problem to these households. There is risk of the roofs being blown off by the wind storm, collapsing of walls and breakage of toilet pots from the falling stones from the rooftops.* —Mason, private sector, Trashigang district, Bhutan

When preventing damage to toilets was not possible, households would need to reconstruct. Barriers to reconstruction included available finances, time, and motivation. Soil type was particularly important for communities that relied on locally available materials for household-constructed pit latrines.

*Because of the soil we have, latrines collapse, these trenches that we dig, when water goes in there, it collapses. We will thus start looking for another place to build another toilet and so we are running out of land.* —Woman, community member, Lua-Luo, Kasama, Zambia

#### Community Ownership and Social Cohesion

Key informants also described the importance of social cohesion or the strength of relationships and the sense of solidarity among community members related to generating demand in rural areas. Similarly, knowledge of the social nature of disease transmission (i.e., if 1 household does not have a latrine, it impacts the whole community) fostered a sense of collective responsibility, which further supported community-led sanitation initiatives and behaviors.

*[Open defecation, OD] practices indirectly affect those with latrines because flies will feed on the feces and land on the meal of those who own latrines. So, those who have latrines must continue to motivate people practicing OD to build a latrine, because sometimes, children drink water directly from the dam which might be contaminated with feces, and this causes waterborne diseases in the community. When people drink that water, they can get cholera or diarrhea, and you know that when cholera infects 10 people in a family, it’s very difficult for them to survive and therefore that’s why we must strive to motivate and educate people on the importance of latrine usage.* —Man, community member, Homa Bay, Kenya

Education for community members on disease transmission was supported by the palpable decrease in disease prevalence over time as community members witnessed improvements in sanitation coverage—allowing for a tangible understanding of the importance of constructing and using latrines. Additionally, collective responsibility, social cohesion, and altruism within communities and between neighbors supported ownership of sanitation coverage at the village level and promoted support for vulnerable individuals in some program areas.

*The most important thing is for them to understand that they are responsible for their health. To maintain sustainability, we try to be there to ensure that whatever we did in the initial phase is maintained but I can’t say that it’s not challenging, it’s challenging.* —Local government staff, Kericho, Kenya

There were definitive differences between contextual factors, including social cohesion, even within countries. For example, social cohesion is generally a very effective facilitator for sanitation programming in African countries, including Kenya and Zambia. Most key informants in both countries reported that community members often help their vulnerable neighbors (including the elderly, orphans, widows, and persons with disabilities) construct latrines. However, select areas visited for this study, including several wards in Kasama district in Zambia, lacked social cohesion and effective traditional leadership due to their periurban nature and rapid population growth as emerging town centers.

*For the rural communities, if we talk about issues of money - some are not able to access money easily. So, the only way they can do that is through my earlier point when I said that the communities themselves should mobilize, once they mobilize themselves, they set laws – the headman should say ‘We all need to have [high-quality] toilets in my community!’ Then, they sit down and they can help each other.* —Local government staff, Kasama, Zambia

### Behavioral Sustainability Factors Around Sanitation Uptake

Behavioral sustainability factors were identified by key informants, especially community members, as barriers or enablers for individual- or household-level decision making related to the construction or use of sanitation facilities. These considerations likely impacted the uptake of sanitation service delivery and were often tied to social or cultural values and norms. Some behavioral sustainability factors included a desire for improved latrines, competing priorities, cultural values of cleanliness, gender roles related to maintenance and cleaning, and knowledge and education about sanitation benefits. Key informants noted that these behavioral factors play an important role in the sustainability of sanitation improvements and should be considered when designing follow-up messaging and sustainable programming.

## DISCUSSION

Sustaining sanitation coverage, defined as access and use of household sanitation facilities, remains a critical challenge in the WASH sector.^6^^,^^26^ This study focused on the identification of factors that likely contributed to the sustainability of sanitation improvements in 4 countries and described how these factors may relate to varying levels of slippage 1–2 years postintervention.^23^ Overall, the sustainability factors identified through this research aligned with findings from previous studies.^16^^–^^18^^,^^20^^,^^26^^–^^29^ To build on existing literature, we also explored the extent to which these factors either facilitated or hindered sustainability within the countries and how service delivery systems either used or addressed the factors based on context. The presence or absence of sustainability factors may have implications on where certain programmatic approaches will work and where adaptations may be required.

According to our data and previous studies, one of the main challenges to achieving sustained sanitation is household poverty—considering both lack of income and lack of affordable sanitation options—which prevents households from constructing high-quality and durable sanitation facilities due to the high cost of materials (e.g., bricks, culverts, and cement) and skilled labor.^26^^,^^27^ Additionally, heavy rains and flooding will frequently result in the collapse of low-quality toilets in areas where households do not have the financial ability to reconstruct, and individuals may lose motivation to prioritize sanitation as a result. However, these barriers may be overcome with the application of interventions targeting social cohesion and sustained behavior change.^27^^–^^30^ We found that in communities with stronger social cohesion, households experiencing poverty reported that their neighbors, community savings groups, religious institutions, or family members would help them construct sanitation facilities. While WASH programs tend to focus on behavioral and individual factors relevant to household-level decision making, studies have shown that communities are often a more critical point of investigation.^31^ The impact of poverty on the sustainability of sanitation improvements was also identified in a concurrent analysis of household survey data that reported that households in the lowest 2 wealth quintiles had higher slippage rates.^23^ Lack of income was less problematic in areas where community members had access to income through fishing, coffee and tea plantations, or farming compounds, some of which were also closer to urban town centers that had a more easily accessible supply of affordable materials.

We found that in communities with stronger social cohesion, households that experienced poverty reported that their community would help them construct sanitation facilities.

In all program areas, a solid foundation for sanitation businesses was established; however, uptake of supply chain improvements by the private sector varied. Supply chain activities completed in all 4 countries aligned with suggestions from existing literature, including mapping of supply chain actors, consumer profiling and willingness-to-pay studies, technical training for masons on improved latrine design, and provision of informed choice materials.^19^^,^^32^^–^^34^ In Kenya, the SAFI (Kiswahili for “clean”) latrine was developed to address gaps in the supply chain after a consumer preference study revealed that appropriate latrine options were limited in the market.^35^ Sanitation businesses were slowly addressing this challenge, and improvements peaked in Homa Bay compared to other areas. In Zambia, sanitation marketing committees (referred to as SanMark) were established to support masons, suppliers, and consumers through training for construction, marketing, and self-financing as well as the development of activities and materials used to promote informed choice.^36^ However, in both Kenya and Zambia, where access to materials can be challenging, supply chain improvements seemed to diminish after SNV was no longer present to support capacity building. In Bhutan and Nepal, where access to materials was not mentioned as readily by participants, supply chain improvements were generally sustained by the private sector, and key informants could easily identify small business owners who supplied sanitation materials (e.g., plumbing, culverts, and cement) in their communities.

Previous research suggests that geographical factors including climate (e.g., heavy rains), natural disasters, soil type, and terrain likely impact the sustainability of sanitation facilities.^17^^,^^18^^,^^27^^,^^29^ However, we also found that the ability of the local service delivery system, including government officials, community volunteers, and masons, to adapt to geographical barriers also played an important role. For example, in Nepal, participants mentioned the challenges of frequent flooding. To address this, masons constructed elevated toilets with doors and roofs that would not fill with water when it rained. Therefore, households were able to maintain their sanitation facilities regardless of flooding. In Suba South in Kenya, collapsible soil from being near Lake Victoria and frequent rains contributed to the frequent collapse of poor-quality pit latrines that likely hindered sustainability, especially in low-income households, as uptake of the SAFI latrine had not yet peaked in this area.^35^

Population density, or the extent to which a village was considered remote, rural, or periurban, also impacted sustainability. In periurban villages in Kasama, Zambia, where slippage was highest, some of the approaches implemented through SSH4A (e.g., community led) seemed no longer applicable as these areas became increasingly urban because of increases in tenancy, less space available for toilet construction, transient populations, and a general lack of community ownership.^27^^,^^30^^,^^37^ According to our data and supported by previous studies, social cohesion and effective local leadership typically support sustained ODF status, especially through community-based behavior change interventions.^27^^,^^30^^,^^37^ We found that community-based demand-creation activities were less effective in periurban areas where tenancy was common and households were close together but not necessarily reliant on each other, therefore lacking social cohesion and community ownership.

Lower-density program areas—including Suba South, Lunte, and Trashigang—also encountered unique challenges that required adaptation, including lack of follow-up visits from health officers and difficulty reaching towns for materials.^15^^,^^38^ Several solutions were identified, including strong traditional leadership in Lunte and improved farm road access in Trashigang. Migratory populations who work as cattle herders (Sakteng, Bhutan) or fishermen (Suba South, Kenya) also required innovative programming as they could not construct toilets while traveling for months at a time. In Suba South, health officers would contact traditional leaders in beach communities to ensure fishermen who were renting houses had access to latrines and to ensure that their families had latrines in their home villages. Some leaders would allow workers to return home for 1 week to construct latrines for their wives and children.

According to our analysis of household survey data, countries with higher levels of sanitation coverage and infrastructure at baseline were less likely to experience slippage of improvements.^23^ Similarly, program areas with large increases in sanitation coverage during the implementation period were more likely to experience slippage compare to those with marginal gains.^23^ According to key informants, the relatively mature sanitation service delivery systems in Asia were more likely to sustain improvements from SSH4A programming compared to the less mature systems in Africa.^25^^,^^26^ For example, in Bhutan and Nepal (where there were higher levels of sanitation coverage at baseline, and accordingly very sustained coverage) respondents reported that they had been using sanitation facilities before the current project was implemented at scale. The investments in supply chain strengthening and promotion of durable toilets were more successful in Bhutan and Nepal where community members were already opposed to open defecation.^22^

Countries with higher levels of sanitation coverage and infrastructure at baseline were less likely to experience slippage of improvements.

Political leadership is considered more influential in driving decisions regarding sanitation service delivery than donors, development agencies, and international organizations.^15^ According to key informants in all 4 countries, the SSH4A approach invested considerable time and resources into strengthening sustainable governance structures through capacity building and technical support. However, the extent to which local governments maintained sufficient financial and human resources for sanitation activities varied between districts and countries. Local government commitment and resource allocation appeared to be more reliable in Nepal and Bhutan compared to Kenya and Zambia. In Nepal, we observed a social movement for sanitation, with all stakeholders prioritizing WASH improvements and motivated to reach ODF status nationwide. Additionally, declaring health as a human right (both in Nepal and Kenya) further strengthened the local government’s role as the sole duty bearer for WASH programs. Collective responsibility among community members and a unique partnership between the local government, local NGOs, and community volunteers led to steep and sustained improvements. In Bhutan, the relationship between the local government, especially at the national level, and SNV was described as exceptionally strong, collaborative, and sustained. All key informants who worked with either the government or SNV mentioned the positive impact of this partnership on the sustainability of sanitation programming. The SSH4A approach is the official approach for sanitation policies within the government of Bhutan, and the national government works closely with SNV and other development partners, which has positive implications for policy development and funding.

We found that program areas with more sustainable sanitation coverage were described as having passionate and innovative local leadership, commitment from the local government, sufficient resources for continuous programming, and the ability to innovate and adapt to contextual challenges, which aligns with existing literature.^39^ Capacity building remained important for sanitation programming after the SSH4A program was complete, including training and engagement led by the local government. Our data suggest that adaptive capacity, which may be impacted by training led by development partner programming; resources allocated for reflection and evaluation, capacity to innovate and respond to challenges, and employee turnover in local health offices, was also related to the maturity of service delivery systems and consequently the sustainability of sanitation programming and coverage. Behavioral enablers, including a household member’s desire for improved sanitation facilities, understanding of the benefits of sanitation, and cultural norms supporting sanitation were found in most program areas, even those that experienced slippage. Even with sustained changes to key sanitation behaviors (which include sharing latrines, using poor quality latrines, and reconstructing after heavy rains), sanitation coverage after the end of project activities relied heavily on governance, supply chain activities, and contextual factors.

Despite sustained changes to key sanitation behaviors, coverage after project activities ended relied heavily on governance, supply chain activities, and contextual factors.

### Strengths and Limitations

Strengths of our research approach included: (1) a wide range of key informants from the community to the national level, which allowed for a comprehensive understanding of sustainability factors; and (2) continuous and extensive conversations and collaboration with SNV staff, which allowed for our analysis to reflect programmatic implications.

An important limitation of this study was the timing of data collection. Interviews were conducted 1–2 years after implementation of the SSH4A approach was completed, and there may have been a lack of understanding or recall bias regarding what was actually implemented. Additionally, FGDs covered a small part of the subnational areas within each country, and components of the SSH4A approach peaked differently in different parts of the countries. The limited number of FGDs may have also hindered the range of opinions and experiences captured in this study.

## PROGRAMMATIC IMPLICATIONS

Findings from our qualitative data, as well as extensive conversations with an advisory group and SNV global and in-country program staff, support the following programmatic implications.

- The maturity of a country’s sanitation service delivery system will likely impact the timeline needed for programming from development partners. In some settings, longer timelines may be needed to strengthen less mature service delivery systems and to build the adaptive capacity in local governments and supply chain systems that is required to sustain sanitation service levels and maintain high coverage.
- The alignment and continuity of efforts to produce sustainable improvements in sanitation service delivery may be affected by the ability to commit to long-term processes from both development organizations (due to short funding cycles) and local leadership (due to political cycles).
- Sustainability-focused support activities—such as advocating for local government involvement, guiding the local government to allocate resources for sanitation over time, supporting reliable monitoring and evidence-based innovation, or agreeing on provision of continuous support with local partners—likely support the longevity of sanitation improvements.
- Reliable monitoring and targeted advocacy should be maintained over a long period to achieve structural change and leverage budgets, and ultimately provide adequate resource allocation for sanitation. Consequently, development organizations may consider implementing sustainability activities after the local government becomes the sole facilitator for sanitation service delivery.

## CONCLUSION

Our data suggest that sustainability factors identified through this study—including poverty, geography, social cohesion, population density, political will, and local government leadership—may have implications on where certain programmatic approaches will work and where adaptations may be required. We found that successful systems strengthening with local government commitment and buy-in coupled with more flexible, responsive, and long-term programming may be required to achieve and maintain long-term access to sanitation. Further research and collaboration with development partners and local governments are needed to more fully understand the impact of these sustainability factors on rural sanitation program design.

## Notes

**Peer Reviewed**

**First published online:** December 13, 2022.
